# Treatment outcomes and prognostic factors of intrahepatic cholangiocarcinoma

**DOI:** 10.3892/or.2013.2290

**Published:** 2013-02-18

**Authors:** RENUMATHY DHANASEKARAN, ALAN W. HEMMING, IVAN ZENDEJAS, THOMAS GEORGE, DAVID R. NELSON, CONSUELO SOLDEVILA-PICO, ROBERTO J. FIRPI, GIUSEPPE MORELLI, VIRGINIA CLARK, RONIEL CABRERA

**Affiliations:** 1 Department of Medicine, University of Florida, Gainesville, FL; 2 Department of Surgery, University of California, San Diego, CA; 3 Department of Surgery, University of Florida, Gainesville, FL; 4 Division of Hematology and Oncology, University of Florida, Gainesville, FL; 5 Division of Gastroenterology, Hepatology and Nutrition, University of Florida, Gainesville, FL, USA

**Keywords:** cholangiocarcinoma, microvascular invasion

## Abstract

The aim of the present study was to determine the treatment outcome and prognostic factors for survival in patients with peripheral intrahepatic cholangiocarcinoma (ICC). A retrospective chart review was performed for patients diagnosed with ICC between 2000 and 2009 at a single institution. We identified a total of 105 patients with ICC. Among them, 63.8% were older than 60 years of age, 50.5% were male and 88.6% were Caucasian. By preoperative imaging approximately half of the patients (50.5%) were surgical candidates and underwent resection. The other half of the patients (49.5%) were unresectable. The unresectable group received chemoradiotherapy (53%) and transarterial chemoembolization (7.7%) as palliative treatments while 23.0% of the patients (12/52) received best supportive care alone. The median survival rates were 16.1 months (13.1–19.2) for the entire cohort, 27.6 months (17.7–37.6) for curative resection, 12.9 months (6.5–19.2) for palliative chemoradiotherapy and 4.9 months (0.4–9.6) for best supportive care (P<0.001). Independent predictors on multivariate analysis were advanced stage at diagnosis and treatment received. In those patients who underwent resection, advanced AJCC stage and presence of microvascular invasion were also independent predictors of poor survival. We concluded that surgery offers the most beneficial curative option and outcome, emphasizing the importance of resectability as a major prognostic factor. The present study also revealed that use of chemoradiotherapy in the adjuvant setting failed to improve survival but its palliative use in those patients with unresectable ICC offered a modest survival advantage over best supportive care. The overriding factors influencing outcome were stage and the presence of microvascular invasion on pathology.

## Introduction

Intrahepatic cholangiocarcinomas (ICCs) are the second most common primary liver malignancy, and they arise from bile duct epithelium within the liver. While 90% of primary liver cancers are hepatocellular carcinomas (HCCs), ICC cases account for the remaining 5–10% (1,2). ICCs are relatively rare malignancies with an age-adjusted incidence rate of 461 per 100,000 individuals reported between 1973 and 2007, as per a recent SEER (Surveillance, Epidemiology and End Results) report (2). While the incidence and mortality of the more common malignancies such as colon, breast and lung are reported to be on a decline nationwide, the incidence of ICC has been on the rise (3–5). In a study based on the SEER database, the incidence and mortality rates of ICC have markedly increased between 1973 and 1997, with an estimated annual percent change (EAPC) of 9.11 and 9.44%, respectively (4).

ICC usually presents sporadically as a discrete intrahepatic mass in patients over 65 years of age (6). These tumors are lethal malignancies with a median survival of 6.5 months from the time of diagnosis, in untreated patients (7). The main risk factor for ICC is primary sclerosing cholangitis (PSC). Recent studies have reported a few additional risk factors including HIV infection, smoking, diabetes, cirrhosis and hepatitis C (HCV) infection (5,8–10). The association between HCV and ICC has gained special attention since the incidences of both are on the rise. A cohort study of a large group of US veterans suggested that HCV infection conferred a >2-fold rise in the risk of ICC (11). However, regardless of etiology, surgical resection offers the only prospect of cure. Yet, only a small percentage of patients with ICC are resectable at the time of diagnosis. For patients with unresectable disease, palliative chemoradiotherapy or palliative ablation/chemoembolization are the major options.

Given the rising incidence of ICC, its poor prognosis and lack of adequate treatment options, further studies clarifying its risk factors, outcomes and prognostic factors are warranted. Most of the existing studies in the US are on surgical patients, and they usually span over a period of decades since this is a rare tumor. A number of questions remain regarding the pathogenesis, criteria for resectability, appropriate adjuvant therapy and palliative therapy. Hence, we conducted a 10-year retrospective study of all patients with ICC treated between 2000 and 2009 at a single institution to evaluate the outcome of various treatments and to assess prognostic factors.

## Patients and methods

The present study was conducted after obtaining approval from the University of Florida Institutional Review Board. Patients diagnosed with ICC who underwent treatment at our institution between 2000 and 2009 were included in our study group. The following data were collected for all patients: age, gender, race, date of diagnosis, presenting complaint, liver function tests at diagnosis, CA 19–9 and CEA levels, stage at diagnosis, presence of metastases, resectability of tumor, date of surgery, pathologic characteristics of tumor, chemotherapy or radiotherapy, recurrence date and site, and date and cause of death.

At the time of diagnosis the tumor stage was assessed by abdominal computed tomography (CT) or abdominal magnetic resonance imaging (MRI) with cholangiopancreatography (MRCP). Preoperative evaluation of vascular involvement was carried out with CT/MRI to determine resectability. In appropriate patients, the tumor was staged by an exploratory laparotomy with intra-operative ultrasound and biopsy. Pre-operative assessment was also performed for extrahepatic primary tumors and metastatic disease. The American Joint Committee on Cancer (AJCC)/International Union Against Cancer (UICC) staging system (7th edition) TNM classification was used for staging the resected tumors in patients who underwent surgery (12).

The treatment modality that each patient received was determined by a multidisciplinary tumor board. Patients did not undergo curative resection if they had metastatic disease at diagnosis, or extensive local vascular invasion or multiple medical comorbidities making them poor surgical candidates. In patients who did not have any of the above, macroscopic curative resection was carried out. Hepatic resection was the main surgery performed, and its extent depended on the segments involved by the tumor. Extrahepatic bile duct resection with Roux-en-Y reconstruction was carried out in patients with bile duct invasion or lymph node metastases. All patients undergoing surgery underwent assessment of lymph node status unless intraperitoneal spread or clear unresectability was identified prior to lymph node assessment. Lymph node dissection included the hepatoduodenal ligament and the porta hepatis. Patients with nodal positivity or positive resection margins were considered to be at high risk for recurrence and were offered adjuvant chemo/radiotherapy. Patients with unresectable tumors were treated with palliative chemotherapy or radiotherapy or transarterial chemoembolization. Some patients declined these treatments and received best supportive care.

Pathologic examination was carried out on all resected tumors. They were examined for tumor size and number, histologic differentiation, and the presence of vascular and perineural invasion. In situations where nodes were dissected, they were examined for the presence of tumor. Surgical margins were examined for the presence of residual tumor. Surrounding liver parenchyma was examined for the presence of changes suggestive of PSC or cirrhosis or other predisposing factors.

After diagnosis, all patients were followed up closely in the clinic. Patients who underwent resection receive regular clinical follow-up along with blood draw for blood chemistry, liver function tests, CA 19-9 level and were screened for recurrence at 6 monthly intervals with CT scans of the chest, abdomen and pelvis.

Study objectives aimed to determine the risk factors, clinical features, treatment outcome and prognostic factors for survival in patients with ICC.

Statistical significance of the difference between the means of quantitative variables was tested using the independent t-test, and the Chi-square test was used for comparing categorical variables. A P-value of 0.05 was held as significant. The Kaplan-Meier method was used to estimate survival, and the duration of survival was derived from the survival curves. Survival of patients in the study group was calculated from the time of diagnosis. Survival curves were compared using the log-rank test. Multivariate analysis was performed using Cox proportional hazards method with backward Wald. Only factors found to be significant on univariate analysis were included in the regression mode, l and a P-value of 0.10 was used to determine if the variable was to be included in the next step.

## Results

Of the 314 patients diagnosed with cholangiocarcinoma during the study period, 105 (33.4%) had intrahepatic tumors. Table I describes the demographic and clinical features of the patients in the study group. An equal percentage of the genders was present (male 50.5%). The majority of patients were older than 40 years (63.8%) and Caucasian (88.6%). The most common presenting complaint was abdominal pain (34.3%), and in a similar percentage of patients the tumor was found incidentally on imaging (30.5%). The tumors arose *de novo* in 82.9% patients without underlying hepatobiliary disease (87/105). Only a minority of the patients had underlying cirrhosis (9.5%) and PSC/UC (6.7%). A majority of patients presented with a solitary tumor (73.3%). The mean tumor size was 5.9 cm (range 1.8–15.0)

**Figure 1:**
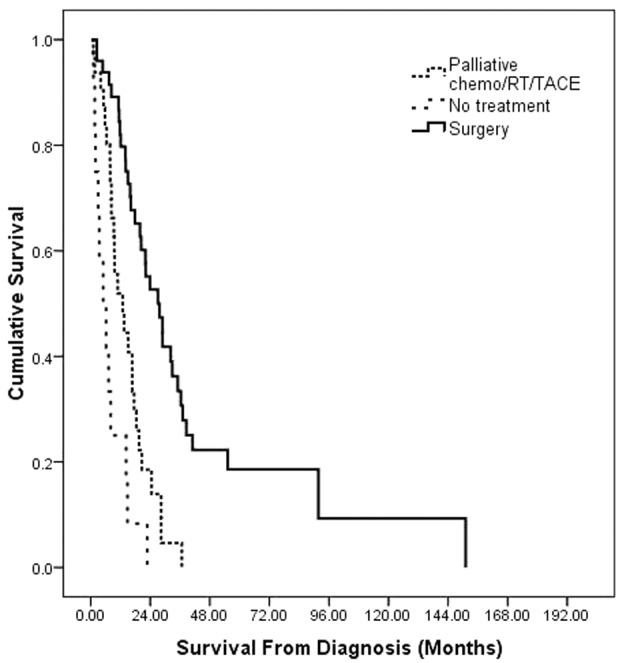
Survival of patients with intrahepatic cholangiocarcinoma based on treatment received.

Median overall survival was 16.1 months (95% CI 13.1–19.2). The survival rates were 63% at 1 year, 17% at 3 years and 9% at 5 years. The median survival after surgical resection was 27.6 months (17.7–37.6), with palliative treatment it was 12.9 months (6.5–19.2) and with best supportive care it was 4.9 months (0.4–9.7) (Fig. 1). Metastatic disease was present at diagnosis in 24 patients (22.9%). The most common sites of metastases were the lung in 8 patients (33.3%) and the peritoneum in 8 patients (33.3%). On univariate analysis, factors associated with survival included tumor stage at diagnosis (P<0.001), total bilirubin >2 mg/dl (P=0.032), presence of metastases at diagnosis (P=0.008) and treatment received (P<0.001) (Table II). Age, gender, ethnicity, albumin <3 gm/dl, underlying risk factors, and elevated CA 19–9 were not associated with survival. On multivariate analysis, advanced stage at diagnosis (P=0.027) and treatment received (P<0.001) were significant independent predictors of survival (Table II).

Of the 105 patients, 53 patients underwent curative surgical resection (50.5%). The type of surgery they underwent was as follows: trisegmentectomy (44.9%), hepatectomy (right 32.6%, left 16.2%) and transplantation (6.1%). The distribution of patients on the basis of their T stage was as follows: T1 (40.8%), T2 (36.7%), T3 (14.3%) and T4 (8.2%). On lymph node dissection 24.5% were node positive. Histopathologic examination revealed the presence of microvascular invasion in 54.7%. The distribution in the different AJCC 7 stages was as follows: I (34.7%), II (18.4%), III (12.2%) and IVa (34.7%). The median survival of patients with stage I was 36.3 months, stage II was 27.1 months, stage III 20.3 months, and stage Iva was 16.1 months (P<0.001). When tumors were classified on the basis of diameter of the largest lesion, 53.1% of tumors were found to be >5 cm. Yet, size was not noted to influence survival when considered as a continuous or as a categorical variable (0.875). Among the components of AJCC staging, nodal positivity was found to most strongly influence staging (P<0.001 on Chi-square test), and the strength of association as assessed by Kendall’s Tau-B for this relationship was 0.753.

**Figure 2:**
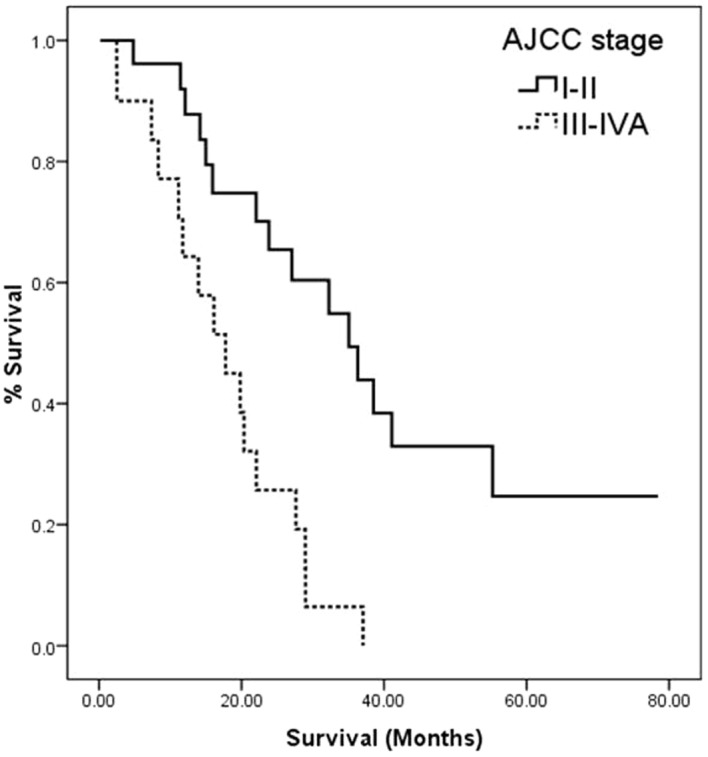
Survival of patients with intrahepatic cholangiocarcinoma who underwent resection stratified by AJCC stage of tumor.

**Figure 3:**
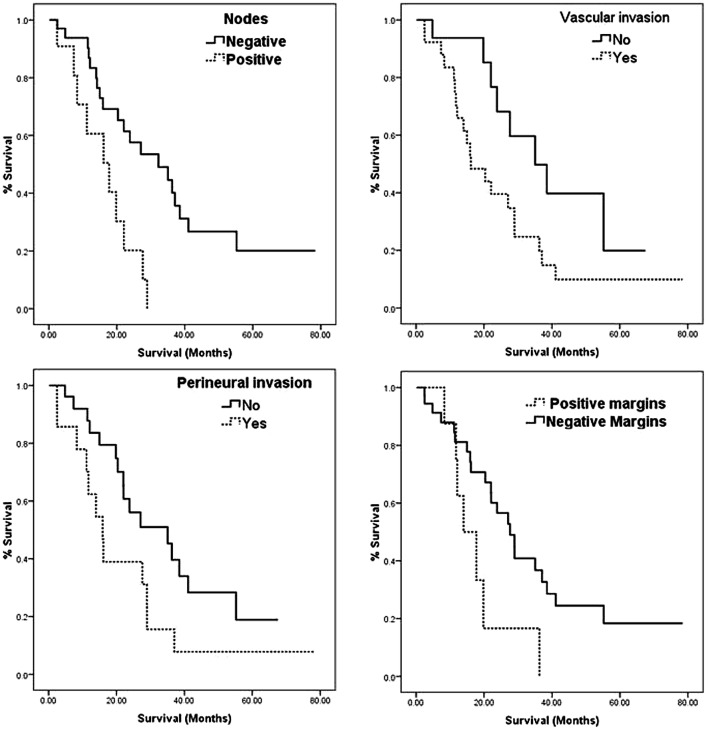
Survival of patients with intrahepatic cholangiocarcinoma who underwent resection stratified by nodal status, microvascular invasion, perineural invasion and margin status.

In 21 patients, surgery was followed by adjuvant chemoradiotherapy (39.6%). The median survival after resection was 27.6 months (17.7–37.6). The survival rates were 82% at 1 year, 33% at 3 years and 19% at 5 years. The median survival in patients with a microscopic negative margin on final pathology (R0 resection) was 28.9 months (22.7–35.1). Table III describes the prognostic factors for survival after resection. On univariate analysis, the AJCC stage of the tumor (P<0.001), the presence of microvascular invasion (P=0.006), perineural invasion (P=0.004), nodal positivity (P=0.002) and positive margins (P=0.028) were factors influencing survival. On multivariate analysis, advanced AJCC stage (P=0.007) and presence of microvascular invasion (P=0.013) were found to be significant and independent predictors of poor survival (Figs. 2 and 3).

After resection, the tumor recurred in 24 patients (45.3%). The mean time to recurrence after resection was 16.7 months (0.7–125.9). The most common site of recurrence was the liver (75%) followed by the lung (25%). AJCC stage, tumor differentiation, microvascular invasion, perineural invasion, nodal positivity, positive margins after resection or adjuvant chemoradiotherapy did not influence the development of recurrence.

Of the overall study group, 52 patients were not eligible for resection (49.5%). The median survival for patients not undergoing resection was 9.5 months (6.3–12.6). The survival rates were 45% at 1 year, 13% at 2 years and 2.6% at 3 years. The reasons for unresectability were the presence of metastatic disease (57.6%), local tumor invasion (28.8%) and presence of comorbidities (11.5%). Palliative chemo/radiotherapy was used in 28 patients (53.8%) and transarterial chemoembolization was performed in 4 patients (7.7%). A minority of patients received best supportive care alone (23.1%). The median survival of patients treated with palliative therapies was 12.9 months (6.5–19.2), while the median survival with best supportive care alone was 5.0 months (0.4–9.6; P=0.007).

## Discussion

The incidence of ICC is on the rise, generating clinical interest in improving outcomes and identifying prognostic factors. In this large series of ICC, we found that only 50% of the patients were resectable based on pre-operative imaging. Furthermore, approximately 60% of those operated on already had local invasion, microvascular invasion or lymph node involvement. Lymph node status was the most important component of staging, stressing the importance of intra-operative lymph node assessment. The most favorable survival was observed in patients operated on with single tumors without vascular invasion or nodal involvement who received R0 resection (with negative surgical margins) (38.5 months vs. 19.8 months). This experience confirms the importance of achieving R0 resection as the key treatment for optimal outcome. The present study also revealed that use of chemoradiotherapy in the adjuvant setting failed to improve survival but its palliative use in those with unresectable ICC offered a modest survival advantage over best supportive care. While the size of the tumor was not a relevant prognostic factor, both stage and surgical treatment independently influenced survival. Furthermore, microvascular invasion on pathology also significantly and independently affected overall survival in patients who underwent resection.

Cholangiocarcinoma is well known to be a cancer more common in older patients (13). In our study, the mean age of patients at the time of ICC diagnosis was 61.8 years, but in those patients with underlying PSC or inflammatory bowel disease, ICC diagnosis occurred at an earlier age, with the mean age at presentation being 53.4 years. While painless jaundice is the most common presenting complaint in patients with hilar or distal cholangiocarcinoma, ICC is known to present more like hepatocellular carcinoma with symptoms of abdominal pain (13–15). Other common presenting symptoms were weight loss and jaundice. However, one third of the patients with ICC were asymptomatic and were incidentally diagnosed upon abdominal imaging. Traditionally, elevated CA 19-9 or high CEA or their combination has been used as diagnostic clues for ICC (14). In our study only about one third of the patients had an elevated CA 19-9 or CEA (36% had a CA 19-9 >100 IU/ml and 30.5% had CEA >2.5 IU/ml) and fewer had an elevation in both, stressing the lack of useful biomarkers in this disease. The lack of specific presenting symptoms, absence of effective tumor markers and the non-existence of screening strategies together often leads to the late diagnosis of cholangiocarcinoma (16).

ICC developed in the background of cirrhosis in close to 10% of the patients, of which the majority (80%) were patients with HCV-related cirrhosis. Epidemiological studies from Eastern nations such as Japan have reported an even higher incidence of HCV infection up to 36% among patients with ICC (1,17–19). A recent large cohort study from the US which included 718,687 veterans also reported a significant association between HCV and ICC (11). HCV RNA has been detected in cholangiocarcinoma cells. This finding indicates that HCV may promote cholangiocyte proliferation and dysplasia of the intrahepatic bile duct epithelium that eventually leads to tumor development (20–22). We were unable to examine the association between HCV and ICC in our data due to the small number of patients with HCV and a lack of a control group.

In our study, 50.5% of patients underwent surgery with curative intent. Curative resection offers the best hope of cure in patients with localized disease. The 5-year survival rate among patients who underwent curative resection was 19% with a median survival of 27.6 months. Previous studies report a variable 5-year survival ranging from 16 to 44% with a median survival rate ranging between 12–59 months (23–31). The AJCC 7th edition staging does not include the size of tumor as a factor for staging tumors when compared to the AJCC 6th edition. Keeping in line with this, we did not find tumor size to influence outcome. Patients who were considered to be at high risk for recurrence due to local invasion and/or positive surgical margins were treated with adjuvant chemoradiotherapy. We did not find a significant survival benefit for patients who received adjuvant chemoradiotherapy over those who did not receive adjuvant therapy (Table III, P=0.174). However, the median survival was slightly longer in the group receiving adjuvant therapy when compared to the group not receiving treatment. The lack of difference in survival probably explains why margin positivity did not turn out to be a prognostic factor on multivariate analysis while AJCC stage and microvascular invasion were found to be predictors of long-term survival after curative resection. Other studies have reported nodal positivity to be an important prognostic factor (27,30,31) yet, this factor was not found to be independently predictive of survival on multivariate analysis. A possible explanation for this finding is the strong correlation between AJCC stage and nodal positivity, as demonstrated by the high Kendall’s Tau-B for this relationship.

After curative resection, the tumor recurred in nearly half of the patients, but the recurrence did not adversely affect long-term survival. Most of the patients who developed recurrence were treated for disease control with palliative chemoembolization or systemic therapy. These interventions probably skewed the survival rates among these patients by eliminating the survival disadvantage one would expect with recurrence. In addition, the mean time to recurrence from surgery was relatively long. In this series established factors such as vascular/perineural invasion or size were not found to predict recurrence. However, most patients with these high-risk factors were treated with adjuvant chemoradiotherapy which probably decreased the chances of recurrence.

In our study, at the time of diagnosis a third of the patients had metastatic disease and about half the patients had unresectable tumors. Only 25% of these patients treated with supportive therapy alone were living at one year from diagnosis. A clear role for the use of palliative treatments in patients with ICC has not been defined. Most studies on palliative chemoradiotherapy concerned patients with hilar or distal cholangiocarcinoma. In this series we showed that palliative radiotherapy/chemotherapy were associated with a more favourable survival when compared with no treatment. Given the rarity of this cancer, prospective randomized clinical trials to confirm this result would be difficult to perform.

Limitations of the study include its small sample size and the long study period during which significant changes in therapy occurred. Yet, among the reported literature for this rare cancer, our study has one of the largest cohorts. While our study included patients treated over the past decade it represents more recent data compared with the older US studies in the literature. Upon our review of the literature (Table IV), these earlier US studies from more than a decade ago had a smaller number of patients and included only patients who underwent resection. The present study presented outcomes for all stages and treatments, including the patients who receive palliative care.

In conclusion, intrahepatic cholangiocarcinomas is associated with poor prognosis and demands improved understanding of the factors that determine development, outcomes and prognosis. In this large series concerning a rare malignancy, surgery offers the most advantageous curative option and outcome, emphasizing the importance of resectability as a major prognostic factor. The present study also revealed that use of chemoradiotherapy in the adjuvant setting failed to improve survival but its palliative use in those with unresectable ICC offered a modest survival advantage over best supportive care. The overriding factors influencing outcome were stage and the presence of microvascular invasion.

## Figures and Tables

**Table I:** Demographic and clinical characteristics of the study group.

Variables	n (%)
Age (years)	
<59	38 (36.20)
≥60	67 (63.80)
Gender	
Male	53 (50.50)
Female	52 (49.50)
Ethnicity	
Caucasian	93 (88.60)
African-American	6 (5.70)
Other	6 (5.70)
Presenting complaint	
Abdominal pain	36 (34.30)
Incidental	32 (30.50)
Painless jaundice	19 (18.10)
Abnormal LFT	7 (6.70)
Weight loss	5 (4.80)
Pain + jaundice	3 (92.90)
High CA 19-9	1 (1.00)
Metastatic disease	1 (1.00)
Risk factor	
None	87 (82.90)
UC + PSC	4 (3.90)
UC	2 (1.90)
Hep C	8 (7.60)
Cirrhosis (non-Hep C)	2 (1.90)
PBC	1 (1.00)
Stage at diagnosis	
I	11 (10.50)
II	23 (21.90)
III	47 (44.80)
IV	24 (22.90)
Tumor focality	
Unifocal	77 (73.30)
Multifocal	18 (17.10)
Presence of metastases at diagnosis	
No	80 (77.10)
Yes	24 (22.90)
Site of metastases	
Lung	8 (7.60)
Peritoneum	8 (7.60)
Liver	4 (3.80)
Bone	2 (1.90)
Other	2 (1.90)
Surgery	
Curative surgery	53 (50.50)
Palliative surgery/staging surgery	12 (11.40)
No surgery	40 (38.10)
Reason for unresectability	
Metastases	30 (28.60)
Local invasion	15 (14.30)
Comorbidities	6 (5.70)
Patient death	1 (1.00)
Radiotherapy	
Adjuvant post-operative	11 (10.50)
Palliative	13 (12.40)
Chemotherapy	
Adjuvant post-operative	18 (17.10)
Palliative	27 (25.70)
Treatment	
Surgery alone	32 (30.50)
Surgery + adjuvant chemo/RT	21 (20.00)
Palliative chemo/RT	28 (26.70)
TACE	4 (3.80)
No treatment	12 (11.40)
Albumin	
<3.0 gm/dl	14 (13.30)
≥3.0 gm/dl	79 (75.20)
Bilirubin	
≥2 mg/dl	22 (21.00)
<2 mg/dl	72 (68.60)
CA 19-9	
≥100 ng/ml	38 (36.20)
<100 mg/ml	67 (63.80)
CEA	
≤2.5 ng/ml	21 (20.00)
>2.5 ng/ml	32 (30.50)
AST	
≤55 IU/ml	48 (45.70)
>55 IU/ml	47 (44.80)
ALT	
≤40 IU/ml	33 (31.40)
>40 IU/ml	62 (59.00)
Alkaline phosphatase	
≤140 IU/ml	40 (38.10)
>140 IU/ml	55 (52.40)

LFT, liver function tests; CA 19-9, carbohydrate antigen 19-9; UC, ulcerative colitis; PSC, primary sclerosing cholangitis; Hep C, hepatitis C; Chemo/RT, chemotherapy/radiotherapy; TACE, transarterial chemoembolization; CEA, carcinoembryonic antigen; AST, aspartate aminotransferase; ALT, alanine aminotransferase.

**Table II:** Univariate and multivariate analysis of factors associated with the survival of all patients with intrahepatic cholangiocarcinoma.

Variables	n	Median survival	95% CI survival	Univariate analysis P-value	Multivariate analysis P-value	HR (95% CI)
Age (years)						
<59	38	17.7	15.5–19.9	0.292		
≥60	67	14.2	11.0–17.5			
Gender						
Male	53	16.6	10.0–23.3	0.822		
Female	52	15.9	12.3–19.5			
Ethnicity						
Caucasian	93	15.9	13.0–18.8	0.141		
African-American	6	22.7	0.0–65.2			
Other	6	14.9	5.6–24.3			
Risk factor						
None	87	15.0	12.0–18.1	0.659		
Cirrhosis	10	23.8	6.4–41.2			
UC/PSC	7	7.1	0.0–34.2			
Stage at diagnosis						
I–II	71	28.9	20.5–37.2	**<0.001**	**0.027**	
III–IV	34	13.5	9.1–17.8			2.0 (1.1–3.7)
Presence of metastases at diagnosis						
No	80	17.7	13.6–21.8	**0.008**	0.204	
Yes	25	9.6	6.2–13.0			1.5 (0.8–2.83)
Treatment						
Surgery	53	27.6	17.6–37.6	**<0.001**	**<0.001**	
Palliative treatment	32	12.8	6.5–19.2			2.2 (1.1–4.1)
Best supportive care	12	4.9	0.3–9.6			5.9 (2.8–12.7)
Albumin						
<3.0 gm/dl	14	14.9	0.0–95.3	0.887		
≥3.0 gm/dl	79	35.0	12.4–17.5			
Bilirubin						
≥2.0 mg/dl	22	9.4	5.7–13.1	**0.032**	0.081	
<2.0 mg/dl	72	16.6	11.2–22.0			0.6 (0.3–1.1)
CA 19-9						
≥100 ng/ml	38	16.6	12.3–20.9	0.991		
<100 mg/ml	67	16.1	12.2–20.0			

UC, ulcerative colitis; PSC, primary sclerosing cholangitis; CA 19-9, carcinoembryonic antigen 19-9. Median survival is expressed in months.

**Table III:** Univariate and multivariate analysis of factors associated with survival of the patients who underwent curative resection.

Variables	n (%)	Median survival	95% CI survival	Univariate analysis P-value	Multivariate analysis P-value	HR (95% CI)
Age (years)						
<59	19 (35.8)	32.2	9.9–54.6	0.386		
≥60	34 (64.2)	27.07	18.1–35.9			
Gender						
Male	29 (54.7)	28.9	21.8–36.0	0.812		
Female	24 (45.3)	20.3	15.4–24.1			
Risk factor						
None	44 (83.0)	27.0	17.2–36.8			
Cirrhosis	5 (9.4)	23.8	10.0–37.6	0.79		
UC/PSC	3 (5.7)	32.2	-			
AJCC stage						
I–II	26 (56.6)	35.1	22.9–47.2	**<0.001**	**0.007**	-
III–IVa	2123 (39.6)	17.7	10.5–24.9			3.1 (1.4–7.0)
Treatment						
Surgery alone	32 (60.4)	23.8	5.0–42.6	0.174		
Surgery + adjuvant chemo/RT	21 (39.6)	27.6	16.9–38.2			
Recurrence						
Yes	24 (45.3)	28.9	16.7–41.2	0.314		
No	29 (54.7)	17.7	0–36.4			
Differentiation						
Well	7 (13.2)	-	-	0.094		
Moderate	31 (58.5)	22.0	9.6–34.5			
Poor	9 (17.0)	28.9	2.6–55.3			
Tumor size (cm)						
<5	18 (33.9)	27.1	19.4–34.7	0.875		
≥5	26 (49.1)	22.0	13.2–30.8			
Microvascular invasion						
Yes	29 (54.7)	16.1	7.8–24.4	**0.006**	**0.013**	-
No	19 (35.8)	38.5	21.4–55.5			3.0 (1.3–7.0)
Perineural invasion						
Yes	17 (32.1)	16.1	12.1–20.0	**0.004**	0.897	-
No	31 (58.5)	35.0	18.8–51.2			0.9 (0.4–2.3)
Surgical margins						
Positive	9 (17.0)	13.9	7.2–20.6	**0.028**	0.131	-
Negative	40 (75.5)	28.9	22.7–35.1			0.5 (0.2–1.3)
Lymph nodes						
Positive	13 (24.5)	17.7	7.8–27.6	**0.002**	0.420	-
Negative	36 (67.9)	35.0	21.3–48.7			1.7 (0.5–5.8)
CA 19-9 level						
<100 ng/ml	16 (30.2)	35.0	19.8–50.2	0.295		
≥100 ng/ml	37 (69.8)	22.0	15.1–29.0			
Albumin level						
≥3.0 gm/dl	39 (73.6)	22.0	13.1–30.9	0.661		
<3.0 gm/dl	8 (15.1)	37.0	33.7–40.3			
Bilirubin level						
<2.0 mg/dl	40 (75.5)	27.6	17.3–37.7	0.816		
≥2.0 mg/dl	7 (13.2)	22.0	12.2–31.8			
NLR						
<5	24 (45.3)	23.8	15.4–32.2	0.349		
≥5	12 (22.6)	32.8	16.6–49.0			

UC, ulcerative colitis; PSC, primary sclerosing cholangitis; AJCC, American Joint Committee on Cancer; Chemo/RT, chemotherapy/radiotherapy; CA 19-9, carbohydrate antigen 19-9; NLR, neutrophil lymphocyte ratio. Median survival is expressed in months.

**Table IV:** Studies on intrahepatic cholangiocarcinoma.

Study (ref.)	Year	Location	Study period	N total/resected	Median survival (months)	Survival rate	Prognostic factors
Chu *et al*(23)	1997	Hong Kong, Philippines	28 years	77	12.2	16%, 5-year	Lymphatic permeation, hilar nodal metastases
Harrison *et al *(24)	1998	USA	26 years	32	59	42%, 5-year	Vascular invasion, satellite lesions
Roayaie *et al*(25)	1998	USA	1991–1997	26/16	42.9	44%, 5-year	Positive margins, tumor size, presence of satellite nodules, degree of tumor necrosis
Leiser *et al*(26)	1998	USA	31 years	61/28	-	60%, 3-year	Tumor stage
Valverde *et al*(27)	1999	France	1990–1997	30	28	22%, 3-year	Satellite nodules, lymph node positivity
Weber *et al*(28)	2001	USA	1992–2000	53/33	37.4	55%, 3-year	Vascular invasion, positive margins, multiple tumors
Ohtsuka *et al*(29)	2002	Japan	1984–2001	62	25.5	23%, 5-year	Multiple tumors, high CA 19-9 levels
Li *et al*(31)	2009	China	1995–2005	136/65	20	20.1%, 5-year	Higher TNM, lymph node metastasis
Guglielmi *et al*(30)	2009	Italy	1990–2007	81/43	40.0	20%, 5-year	Macroscopic tumor type, lymph node, metastases, vascular invasion
